# An Unusual Perioral Presentation of Pityriasis Folliculorum Mimicking Periorificial Dermatitis

**DOI:** 10.7759/cureus.112929

**Published:** 2026-07-18

**Authors:** Hanna Kunzler, Lillian Sun, Bethany R Rohr

**Affiliations:** 1 Dermatology, Ohio University Heritage College of Osteopathic Medicine, Cleveland, USA; 2 Dermatology, University Hospitals Cleveland Medical Center, Cleveland, USA

**Keywords:** demodex mites, hypopigmentation, inflammatory dermatoses, ivermectin, nutmeg grater, perioral rash, pityriasis folliculorum, topical crotamiton, topical permethrin, topical tretinoin

## Abstract

*Demodex* mites are commonly found on human skin, but excessive proliferation can contribute to inflammatory eruptions. Pityriasis folliculorum is a *Demodex*‑associated condition characterized by follicular roughness and fine scale. Although it most often affects the cheeks, its presentation can vary and may resemble other papulopustular disorders.

We describe a patient who developed a perioral papulopustular eruption with prominent follicular roughness. The condition persisted despite treatment with topical metronidazole, topical tretinoin, and topical antifungal therapy. Given the lack of response to topical therapies, a potassium hydroxide (KOH) skin scraping was performed and demonstrated numerous *Demodex* mites, with rapid resolution following the initiation of topical ivermectin.

This case highlights the importance of considering *Demodex*‑related disease in the evaluation of perioral papulopustular eruptions when standard therapies are ineffective. Early recognition can help guide appropriate management and prevent prolonged symptoms.

## Introduction

While *Demodex* mites are considered a normal part of the skin microbiome, demodicosis results when there is an overgrowth of these organisms. Demodicosis is diagnosed when there are >5 *Demodex* mites/cm^2 ^[1]. *Demodex folliculorum* is commonly found in sebaceous areas, typically in the infundibular portion of hair follicles, with an increase in population with increased host age [2]. Clinically, demodicosis includes a spectrum of disease ranging from asymptomatic overgrowth to inflammatory phenotypes. This spectrum includes pityriasis folliculorum, which is typically characterized by follicular plugging and fine white scale and may progress toward the broader rosacea spectrum [1,3]. Demodicosis has been associated with many inflammatory dermatoses, such as folliculitis, acne, seborrheic dermatitis, and rosacea [1,3]. Pityriasis folliculorum may be a subtype of rosacea caused by *Demodex* overgrowth that presents on the cheeks with follicular plugging and fine white scale, producing a "nutmeg grater" appearance [4]. We present a case of pityriasis folliculorum with a perioral dermatitis-like presentation that was resistant to topical metronidazole, topical tretinoin, and topical antifungal treatment but resolved quickly with topical ivermectin. This case emphasizes the importance of considering pityriasis folliculorum in the differential diagnosis of perioral papular or scaly eruptions, particularly when standard therapies fail.

## Case presentation

A healthy, 45-year-old African American woman presented with a two-year history of a perioral eruption. Prior outside treatment included topical metronidazole cream for an unclear duration without benefit. The patient has Fitzpatrick skin type 5 and no known triggering factors. She had scattered hypopigmented macules coalescing into small patches and pinpoint hypopigmented papules in a perioral distribution (Figure 1).

**Figure 1:**
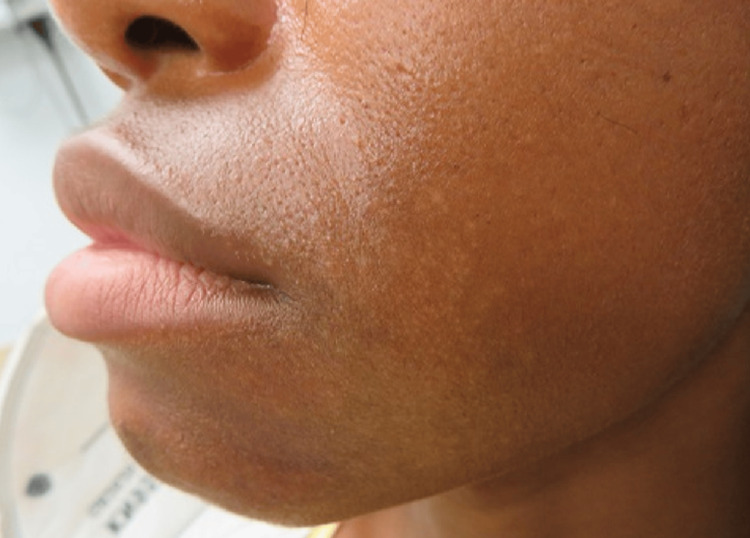
The patient first presented with scattered hypopigmented macules coalescing into small patches and pinpoint hypopigmented papules in a perioral distribution.

The favored diagnosis was periorificial dermatitis with post-inflammatory hypopigmentation. Topical metronidazole was restarted to treat the dermatitis, and topical tretinoin 0.025% cream and daily sunscreen were added to address the post-inflammatory hypopigmentation. The patient reported no improvement after three months of compliant treatment. At the follow-up visit, she was not wearing moisturizer, and her examination was newly notable for fine white scale with pinpoint papules in a perioral distribution (Figure 2). A potassium hydroxide (KOH) preparation interpreted by a dermatologist revealed areas of fungal elements suspicious for short hyphae and spores, suggesting the presence of *Malassezia* species. The patient was treated with antifungal shampoo as a face wash once weekly and ketoconazole 2% cream twice daily to the affected area for four months, but this resulted in minimal improvement. At follow-up, there were fine hypopigmented papules and macules with overlying fine white scale in a perioral distribution (Figure 2).

**Figure 2:**
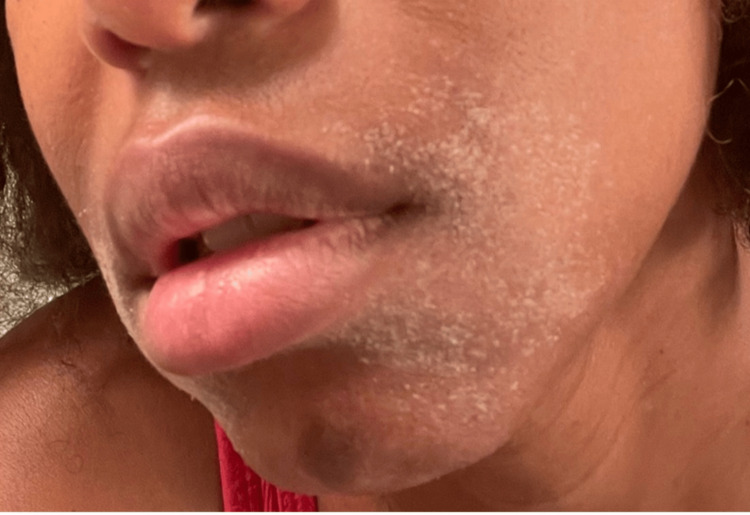
At the follow-up visit, the patient was not wearing moisturizer, and her examination was significant for fine white scale with pinpoint papules in a perioral distribution. Of note, the brown hyperpigmented patch on her central chin is from a prior burn and not related to her rash.

A repeat KOH preparation with a 15 blade was performed with more attention to scraping a discreet small papule. The examination of the skin scraping revealed numerous (>8) *Demodex* mites (Figure 3).

**Figure 3:**
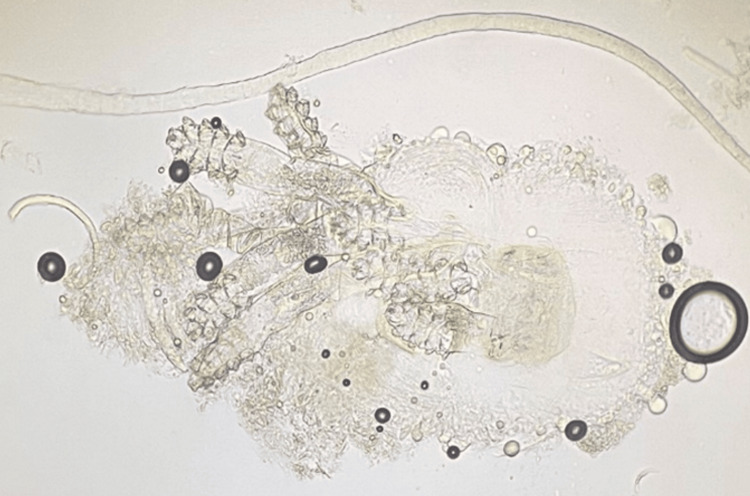
KOH preparation of skin scraping from a discrete follicular papule on the face revealed >8 Demodex mites per high-power field at 400× magnification. KOH: potassium hydroxide

A diagnosis of pityriasis folliculorum was made based on the >8 *Demodex* mites on skin scraping with KOH preparation, and the patient was started on ivermectin 1% cream once daily to the affected areas. She was continued on topical tretinoin and sun-protective measures to address post-inflammatory changes. Three months later, the small papules and overlying scale had completely resolved, and post-inflammatory hypopigmentation had improved (Figure 4).

**Figure 4:**
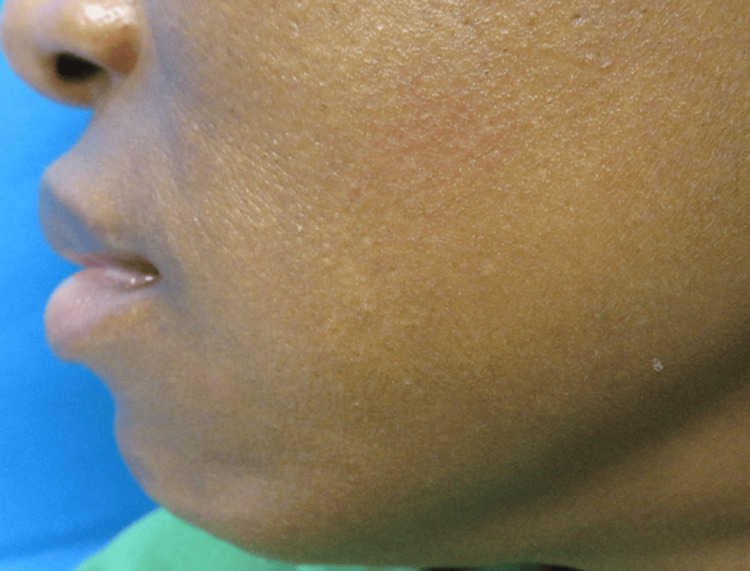
After treatment with ivermectin 1% cream for three months, the patient's symptoms had resolved.

The frequency of the ivermectin cream was decreased to every other day for maintenance, and topical tretinoin was continued.

This was a case of pityriasis folliculorum with a perioral dermatitis and seborrhea-like presentation that was resistant to topical metronidazole, topical tretinoin, and topical antifungal treatment but resolved quickly with topical ivermectin. While *Demodex* mites are considered a normal part of the skin microbiome, demodicosis results when there is an overgrowth of these organisms (>5 *Demodex* mites/cm^2^) [1]. Demodicosis has been associated with many inflammatory dermatoses, such as rosacea [1,3]. Pityriasis folliculorum may be a subtype of rosacea caused by *Demodex* overgrowth that presents on the cheeks with follicular plugging and fine white scale, producing a "nutmeg grater" appearance [4]. Increased *Demodex* density is thought to drive rosacea pathogenesis by disrupting host innate immune surveillance, triggering host inflammatory cascades, follicular obstruction, and the facilitation of bacterial proliferation within the pilosebaceous subunit [5].

## Discussion

Clinical findings and therapeutic response in this case supported a diagnosis of *Demodex*-associated disease. The fine white "nutmeg grater" scale was a key morphologic feature that had been camouflaged by the patient's moisturizer on the original consultation. When evaluating for *Demodex* overgrowth, it is important to focus on scraping discrete papules or pustules that are present, as the diagnosis may be missed otherwise. While our patient responded to topical ivermectin, other treatment options include topical sulfur products, topical permethrin, topical crotamiton, oral ivermectin, and oral metronidazole [3,6].

Pityriasis folliculorum typically manifests as small follicular papules and pustules distributed across the central face, often accompanied by erythema and a roughened, sandpaper-like texture. It represents a stage of *Demodex* proliferation characterized by minimal host inflammatory response and is frequently regarded as a preliminary phase of papulopustular rosacea, in which widespread *Demodex* overgrowth occurs alongside a diminished immune reaction [7]. A systematic review and meta-analysis investigating the global prevalence of *Demodex* in humans concluded that men and women have a similar prevalence of *Demodex* (38% in women and 37% in men) [8]. In contrast, perioral dermatitis presents with discrete inflammatory papules and pustules in a more localized distribution, classically confined to the nasolabial folds and chin while sparing the vermilion border [9]. Although perioral dermatitis can share papulopustular morphology with pityriasis folliculorum, its etiology is complex and not thought to be primarily driven by *Demodex* proliferation [9].

Another distinctive feature of our case is the presence of hypopigmentation, an uncommon manifestation of *Demodex*‑associated disease. Although pigmentary alteration is rarely emphasized in the demodicosis literature, emerging evidence suggests that *Demodex* overgrowth may contribute to atypical pigmentary changes. A cohort study of patients with Fitzpatrick skin types 3-4 demonstrated that pigmented demodicosis, while uncommon, can be an overlooked cause of facial hyperpigmentation [10].

These findings suggest that *Demodex*‑associated disease may contribute to a wider range of pigmentary changes than traditionally recognized, although this possibility remains preliminary and based on limited observation. In our patient with Fitzpatrick skin type 5, hypopigmentation was noted, but its relationship to *Demodex* overgrowth requires further study before broader conclusions can be drawn. The recognition of potential pigmentary variability remains important in skin of color, where such changes can be more pronounced and diagnostically misleading. The increasing awareness of these atypical presentations may help dermatologists more effectively identify and manage *Demodex*‑associated disease across diverse skin types.

Our case was unique in that the patient initially presented with hypopigmented macules coalescing into small patches and pinpoint hypopigmented papules in a perioral distribution, favoring a diagnosis of periorificial dermatitis with post-inflammatory hypopigmentation. However, the minimal response to topical metronidazole therapy, combined with the subsequent KOH preparation revealing numerous *Demodex* organisms, ultimately supported the diagnosis of pityriasis folliculorum.

## Conclusions

This case underscores the importance of reconsidering the diagnosis in persistent facial eruptions and recognizing *Demodex*-associated disease as a potential mimic of periorificial dermatitis, particularly when characteristic follicular scale is present and conventional therapies fail. Dermatologists should remain attentive to overlapping clinical features between pityriasis folliculorum and perioral dermatitis, as accurate differentiation has direct implications for treatment strategy and patient outcomes. Although hypopigmentation has been observed clinically in this case of suspected *Demodex* overgrowth, its relationship to demodicosis remains speculative, and current evidence more clearly supports associations with hyperpigmentation. Further investigation is needed to clarify these pigmentary changes. Ultimately, maintaining a broad differential and incorporating targeted diagnostic testing can prevent prolonged morbidity and ensure timely and effective intervention.
